# “One of Us”: Reframed Labels, Compassion and Hope in Voluntary Prison Work With Ex-Servicemen

**DOI:** 10.1177/0306624X221124838

**Published:** 2022-10-08

**Authors:** Katie Salt, Zetta G. Kougiali

**Affiliations:** 1 University of East London, UK; 2 University of Roehampton, London, UK

**Keywords:** prisons, penal voluntary sector, ex-Armed Forces, IPA

## Abstract

Despite the growing body of literature on Prison Officers and therapeutic practitioners within correctional facilities, comparatively little research exists into prison volunteers. Using semi-structured interviews with caseworkers (*n* = 5), analyzed via Interpretative Phenomenological Analysis, this study explores the experience of being a Prison In-Reach Caseworker, supporting the male ex-Armed Forces population in Greater London prisons. Through identifying three superordinate themes of the inherent moral values of the shared past, compassion and “in” versus “out” of the prison system, the study concludes that the caseworkers, working outside the boundaries of the correctional system, reject the label of “criminal” and its associated consequences, choosing instead to attribute value and dignity to the prisoners, both as ex-Armed Forces personnel, and as human beings. The findings offer an insight into the consequences of positive labeling for perspectives of redemption and desistance and suggest the need for further investigation into the experiences and impact of prison volunteers working with different populations.

## Introduction

The limited evidence of prison effectiveness in reducing recidivism, as well as the increasing tendency toward risk management approaches, has been repeatedly noted in recent literature (see, e.g., Crawley, 2004, p. 96; Cullen et al., 2011; Prison Reform Trust, 2018). Indeed, the suggestion that prison is not only ineffective but can have potentially criminogenic effects (Cullen et al., 2011) has been made alongside the realization that “only when people are treated like fellow citizens are they likely to behave accordingly” (Gregory et al., 2006, p. 204). As a consequence, the development of therapeutic interventions such as the Good Lives Model (Ward, 2002), which suggests that the safer societies are achieved by assisting offenders to adopt more fulfilling and socially integrated lifestyles, has marked the start of a significant departure from risk management practices, traditionally employed in correctional settings, toward strengths-based approaches.

Psychological paradigms and discourses that focus on criminal personalities and assume offenders’ pathology have been criticized as ineffective (Fox, 1999 ), while those capitalizing on positive relationships between practitioners and offenders have been recently recommended (Robertson et al., 2011). In recognition of the critical role of those who choose to work on the frontline of correctional services, therapeutic practitioners, as well as Prison Officers, have become the focus of a handful of exploratory studies (see e.g., Bennett et al., 2008; Crawley, 2004; Harvey & Smedley, 2010; Liebling et al., 2012). Many Prison Officers perceive their experience as misrepresented and misunderstood (Smith, 2016), and Crawley (2004) highlights that they are a population about whom “we know astonishingly little, despite the fact that [they] typically spend a far higher proportion of their lives in prison than do many of their charges” (p. xiii). With terms such as “offender” having become institutionalized to such an extent that the people who bear its label are deemed “the most marginalized and potentially dangerous members of society” (Gorman et al., 2006, p. 26), it is important to understand the experiences of individuals who choose to spend their time with people “whom most of us would be both frightened and disgusted to be near” (Dilulio, 1987, p. 169), and in institutions in which most people are held against their will. Moreover, with the ever-increasing prison population (Prison Reform Trust, 2018) and the Prison Service having acknowledged the stressful conditions of prison work (Crawley, 2004, p. 38), this is a topic that can only become more relevant with time.

Against this background of radical change in the perception of “offenders,” and critical examination of those who work with them, there remains a thriving population about whom there is a dearth of in-depth experiential research: prison volunteers. Volunteers and voluntary organizations are heavily relied upon in the criminal justice system worldwide (Tomczak, 2017) for the care and treatment of imprisoned individuals as decreasing budgets and resource limitations cannot be covered by staff alone (Federal Bureau of Prisons 2009, 2010 ; Kort-Butler & Malone, 2015). It is, perhaps, impossible to know the exact number of people throughout England alone who choose to undertake this role, but research about this population and their oft-cited “distinctive and valuable approach” (Tomczak & Albertson, 2016, p. 59; see also Crewe et al., 2014) is conspicuous by its absence. Considering what we know about Prison Officers, many of whom feel “unvalued, undirected and unsure of their role” (Crawley, 2004, p. 4) and are, in fact, “leaving prisons almost at the same rate that they are joining” (The Howard League, 2017, p. 2), this seems a notable omission, especially as prominent third sector organizations are keen to see “more volunteers in our prisons” (Clinks, 2016, p. 2) and thus must be able to attract, and retain them.

Although, of course, the relationship between voluntary sector *organizations* and the Prison Service has been documented in part, this has, to date, been almost exclusively focused on the practicalities of these partnerships and the costs and benefits of outsourcing rehabilitation services for both sides (e.g., Mills et al., 2012; Neilson, 2009). In contrast, little attention has been paid to the understanding that relationships between *individual* volunteers and prisoners can both affect the experience of imprisonment as it is happening (Crewe et al., 2014) and, fundamentally, be transformational for the prisoners’ future. While Tomczak and Albertson (2016) have suggested this is due, in part, to the compassionate approach taken by volunteers, which centers around remaining non-judgmental and working in conjunction with the prisoner to facilitate change, the experience of the volunteers—the reasons behind this approach, and how it makes them feel—has been largely neglected in favor of understanding its impact on the prisoners themselves.

Compassionate behavior can have a positive impact and make the carceral experience gentler (Liebling, 2004), and volunteers are often contrasted with statutory staff as their role allows them to employ non-judgmental and non-punitive working styles that contradict the risk management focus of correctional facilities (Maguire, 2012; Tomczak, 2017). Volunteers’ non-authoritarian approach is facilitated by the limits of their role: as non-statutory staff, they can perform outside managerial pressures and objectives. However, while such roles allow more compassionate practices to be implemented, they also involve being at the receiving end of challenging and often distressing information while having limited, if any, input in the decision making process of the Prison System and the treatment of prisoners (Tomczak & Bennett, 2020). Jacobi and Roberts (2016) discuss the primary and secondary trauma in prison volunteers and the “compassions fatigue” induced by exposure to stories of trauma and loss. More recently, Quinn et al. (2022) highlight the emotional toll of this role by discussing the emotional strategies employed by penal voluntary sector practitioners to mitigate overwhelming experiences and continue supporting criminalized people amidst chronic resource shortages and systemic failures. Considering the above, it is essential to examine volunteer work as performed within broader structures and systemic pressures that contribute to this role’s complex and, often traumatic, emotional landscape.

The Howard League (2011) asserts that ex-service personnel represent, by occupation, the largest subset of the adult male prison population and, indeed, there are currently thousands of voluntary sector agencies delivering support to ex-servicemen in and out of prison (Albertson et al., 2015). Recent reports indicate that although a military background does not increase the likelihood of involvement with the Criminal Justice System, former Armed Forces personnel are twice as likely to be convicted of a sex offense (Howard League, 2011) and three times more likely to be convicted of a violent offense than the general public (MacManus et al., 2013). It is important to note that these findings should be interpreted within the boundaries of the clinical complexity recognized in ex-military personnel due to a combination of adverse life experiences and comorbidities. Early childhood difficulties (Iversen et al., 2007 ), depression, anxiety, substance or alcohol misuse (Graham & Livingston, 2011; Thomas et al., 2010), as well as the psychological and emotional impact of war, often understood as trauma and PTSD, being marginalized out of civilian society and struggling to reintegrate, present a unique set of experiences that can place this population at risk of offending and re-offending (Murray, 2014). The contradictory perceptions and connotations of the term “veteran” within the Criminal Justice system are also of importance, as the social identity of ex-military personnel in prison encompasses both the stigma of the “offender” having committed a crime and, simultaneously, the pride from their patriotic contributions and having done “good” (Murray, 2014).

Veterans who return to civilian life have been noted to demonstrate high levels of distress and difficulties in bridging the conflict between their military identity and values of self-sacrifice, duty, and honor with individualistic and materialistic civic values. Demers (2011) reports that veterans struggle to reconnect with family and friends, perceive a sense of difference and “otherness” and inability to find common ground in discussions with civilians who appear to lack an understanding of their military past. Such feelings of difference and the challenges associated with employing a new identity appear to be diminished in supportive social interactions, especially with other veterans (Demers, 2011; Hunt & Robbins, 2001; Tick, 2005). Shared narratives of common past experiences and values as negotiated within peer networks have been documented as crucial in navigating the transition to a civilian way of life for veterans (Keeling, 2018). The positive outcomes of shared narratives have been extensively documented in the literature and observed in various peer groups and networks in the community (e.g., Cain, 1991; Steffen, 1997) and in prison settings (Kougiali et al., 2019).

The research outlined in this section demonstrates the importance of work undertaken by volunteers within the limitations and stressors induced by the challenging structures they occupy. We have also noted the prevalence and unique characteristics of veterans within the Criminal Justice System, the benefits of social support, and the value of shared experience in reintegration. Despite the beneficial effects of social support, aligning with the distinct “compassion-focused” approach employed by volunteers, there is limited examination of their lived realities, especially when working with veterans. To address this gap, we focus on an in-depth exploration of the lived experience of volunteers supporting imprisoned ex-Armed Forces personnel, a population that has long been identified as having a unique set of experiences and characteristics.

## Materials and Methods

### Procedures

The participants were recruited after responding to an open invitation disseminated, along with the participant invitation letter detailing the nature and purpose of the research, by the Prison In-Reach Casework branch Divisional Secretary on behalf of the researcher to all Greater London Prison In-Reach Caseworkers. The researcher’s sample was naturally limited to those caseworkers who felt able to participate in the research; the number of participants was in line with methodological and epistemological suggestions of interviewing 5 to 10 participants who have all experienced similar events and phenomena when employing phenomenological approaches (see Braun & Clarke, 2021; Polkinghorne, 1989). A semi-structured interview method of data collection was chosen; this approach allows for the exploration of the lived experiences of participants in a way that makes sense to them and, as a method performed in interaction, can potentially allow for new explanations and aspects of the experience to be considered and generated during the interviews (Curtis & Curtis, 2011).

The semi-structured, one-to-one interviews lasted between 45 and 60 minutes at a mutually agreed time and place; four of the interviews took place via telephone and one face-to-face in the researcher’s otherwise unoccupied office. The interviews were audio-recorded, transcribed verbatim, and each participant was given a male pseudonym; the transcripts were then subjected to Interpretative Phenomenological Analysis.

The participants, Charlie, James, Matthew, Paul and Richard (pseudonyms are used throughout), identified as current Prison In-Reach Caseworkers, volunteering as the sole caseworker within one or more Greater London adult male prisons; all participants were recruited from the same charitable organization. The organization provides support exclusively to ex-British Armed Forces personnel in and out of prison, and therefore the caseworkers’ remit is limited to verified ex-servicemen in prison (i.e., prisoners who have been independently confirmed as having one or more days of military service). At the time of the interviews, four of the caseworkers were retired, one was in full-time employment alongside his role, and three had declared personal or familial experience in the Armed Forces. None of the caseworkers had any personal or familial experience of imprisonment.

### Analytical Method

Interpretative Phenomenological Analysis (IPA) is an experiential form of qualitative data analysis that aims to construct a detailed understanding of the meaning of a phenomenon as experienced, understood and made sense of at an individual level (Smith, 2011). It is meticulously idiographic in nature, concerned with understanding the nuance of a distinct experience through exploring it from a distinct perspective in a distinct context. For this reason, it has been argued, it is dependent on going “back to the things themselves” (Husserl, 2001, p. 168) as far as possible: the depth of lived experience as it is presented, without preconception. This suggests that IPA is built on the theoretical foundations of phenomenology and symbolic interactionism, which holds that “human beings are not passive perceivers of an objective reality” (Brocki & Wearden, 2006, p. 3) but rather construct subjective reports that help them to interpret and understand their world (Shinebourne, 2011). However, it is also fundamental to note that *other people’s* experiences (as opposed to one’s own) are at the heart of this method; thus, the concept of interpretation—or hermeneutics—is equally important (Grondin, 1994). Indeed, it has been argued that “without the phenomenology, there would be nothing to interpret; without the hermeneutics, the phenomenon would not be seen” (Smith et al., 2009, p. 37), suggesting that interpretation is the key pathway into understanding. Given the researcher’s personal experience of prison in-reach casework and IPA concerning “topics which are complex, ambiguous and emotionally laden” (Smith & Osborn, 2015, p. 41), it is both an appropriate epistemology and a unique opportunity to interpret the experiences of other Prison In-Reach Caseworkers through the lens of shared experience.

It is, therefore, important to acknowledge that IPA is particularly susceptible to the influence of personal context, particularly as the researcher’s role can be seen as “trying to make sense of the participant trying to make sense of what is happening to them” (Smith et al., 2009, p. 3), thus invoking a double hermeneutic. Therefore, IPA requires a degree of reflexivity—an ability to evaluate oneself and one’s preconceived notions about the research area—followed by a conscious process of “bracketing” off these preconceptions and biases, including those resulting from an awareness of previous research or personal experience, so that they do not obscure the true narrative (Husserl, 1983). For this research, then, when reading and engaging with the caseworkers’ transcript data, the researcher was mindful to suspend, as far as practicable, their presuppositions and judgments about their own experiences of being a Prison In-Reach Caseworker in a Greater London prison, including any similarities and differences in terms of individual encounters with prisoners and Prison Officers, and engage in a process of continual reflexivity and meaning-checking against the caseworkers’ own words. Although, of course, IPA necessitates the researcher’s own interpretation, and indeed the issue of bracketing *per se* is contentious (see, e.g., Gearing, 2004; Heidegger, 1962), this conscious process was, nevertheless, of fundamental importance during the transcript analysis to ensure that the caseworkers’ own experiences, rather than the researcher’s perspective, were reflected in the narrative.

Practically, the data analysis process involved iterative transcript annotation, first using basic note-taking to focus on “interesting or significant” elements in each of the caseworkers’ descriptions and recording these in the left-hand margins (Smith & Osborn, 2008, p. 67). Then, entered in the right-hand margins, related elements of the transcripts were identified through an assessment of the notes to develop potential theme titles, “concise phrases which aim to capture the essential quality of what was found in the text” (Smith & Osborn, 2008, p. 68) as interpreted by the researcher, which sought to illustrate central tenets of the caseworkers’ experience as described. Finally, these potential theme titles were collected into overarching clusters of themes, called superordinate themes, grouped in a table alongside related transcript extracts and continually refined, with constant reference to the caseworkers’ own words.

### Ethical Considerations

This research was granted ethical approval by the host University. As fundamental ethical considerations, all participants were over 18 years of age and were fully informed of the intention of the research before agreeing to participate. Participants were invited to read, digest and question the nature and purpose of the research ahead of consenting and were debriefed after the end of the interviews.

A further imperative when conducting research is “acting with integrity,” which includes “being honest, truthful, accurate and consistent in one’s actions, words, decisions, methods and outcomes” (The British Psychological Society, 2018, p. 7), including recognizing the potential for the relationship between the interviewer and interviewee to influence participant responses. This was particularly pertinent given the researcher’s role as a Prison In-Reach Caseworker alongside the participants, which participants were reminded of throughout. It is, perhaps, useful here to note that the researcher is employed full-time, independent of their role as a caseworker, and has no personal experience of, or connection with, the Armed Forces, past a profound interest in the impact of the institution on the individuals within it. The potential impact of this knowledge on the participants’ answers was addressed through the deliberate use of open questions in order to enable participants to set the parameters of the discussion and the researcher’s conscious effort to put personal experiences and perceptions of casework to one side both during the interviews and subsequent interpretation. As Interpretative Phenomenological Analysis requires both active and subjective interpretation by the researcher and is, therefore, particularly susceptible to the influence of personal experience or bias, this was essential to ensure that the resulting narrative represented a true reflection of the caseworkers’ experiences.

## Results

Through applying the principles of Interpretative Phenomenological Analysis (IPA) to the interview transcripts, three superordinate themes and two subordinate themes were identified, which encapsulate how Prison In-Reach Caseworkers experience and perceive their role: the inherent moral values of the shared past, compassion and “in” versus “out” of the prison system. These themes are captured in Table 1.

**Table 1.** Superordinate and Subordinate Themes.

Superordinate themes	Subordinate themes
1. The inherent moral values of the shared past	1a. Belief in the innate potential for goodness in the prisoners
	1b. Crime as a mistake or misfortune
2. Compassion and the shared obligation to support ex-servicemen in prison	
3. “In” versus “out” of the prison system	

### The Inherent Moral Values of the Shared Past

The first theme that was identified during the examination of the transcripts was the importance of the prisoners’ past as ex-servicemen, which conferred beliefs about their moral value systems, differentiated them from other individuals who offend and was regarded as a defining set of virtues which would both justify their crimes and strengthen their potential to change.

#### Belief in the innate potential for goodness in the prisoners

The transcripts revealed a collective sense among the caseworkers that there is an inherent capacity within the prisoners to achieve “goodness.” For some of the caseworkers, this potential is demonstrated through the prisoners’ identification as ex-servicemen, something, for Charlie, which sets them apart from “the man-on-the-street type criminal”:

*I wasn’t actually interested in just visiting prisons to help prisoners but when [the organization] began doing, um, Prison In-Reach for, er, former serving, services people I thought, well, there’s obviously got to be something good about them or they wouldn’t have been in the services in the first place, so there would have been a base there of a level of discipline and um, of you know, doing something for your country.*

Charlie’s explicit differentiation of “prisoners” and “former serving, services people” reveals differential preconceptions between prisoners: he considers ex-servicemen as a distinct group, separated from other prisoners because there is “something good” about them, despite their imprisonment. Charlie’s confidence that this “obvious” “goodness” stems from “doing something for your country” suggests that it is important for him to establish and maintain this conceptual separation: after all, imprisonment can be understood as a country’s punishment for people who have done something *against* it, by breaking its law(s). Through elevating ex-servicemen above other prisoners, Charlie can succeed in holding two contradictory perspectives (ex-servicemen are “good,” whereas “prisoners” are not) and, as a result, rationalize his decision to “help [the] prisoners” who he feels have proven their “goodness” before—and might do so again. Paul, too, acknowledges that “I couldn’t imagine if I was in a prison otherwise, um, who else, what sort of group I’d be dealing with,” which suggests that he shares Charlie’s belief that ex-servicemen are somehow different and, it can be inferred, superior, compared to others in the prison population. The use of the word “group” here is also indicative of an instinctive and desirable compartmentalization of the prisoners: defining a group also defines those who are excluded from that group and, in Paul’s case, offers the opportunity for him to “imagine” those excluded as different and, it follows, “worse” than the ex-servicemen. Paul’s conceptual exchange of the label “prisoner” for “ex-serviceman” allows him to recognize and group “ex-servicemen” by their virtues instead of their failings, thereby minimizing the significance of their imprisonment and reinforcing his belief in their potential for goodness.

It is important to recognize that all caseworkers categorize ex-servicemen by their membership of the Armed Forces in general terms, drawing no distinction between the different branches, length of service or superiority of rank: at no point did the caseworkers question this categorization or attempt to define its parameters. This suggests that the caseworkers all consider military membership itself to be demonstrative of an individual’s character and, therefore, are able to ascribe “goodness” as a collective attribute to those who have served. Being able to recognize this, in turn, allows the caseworkers to attribute worth to their role: as Matthew notes, when talking about his motivations to help, “you’re doing it because you think it’s worthwhile and, and because you, er, they were in the services and therefore, er, have that value nationally.” With a distinguished career in the military himself, Matthew’s use of “you/ they” assumes the generalized true “value” of any military man, perhaps indicative of his ability to relate to the ex-servicemen because of their shared experience. Indeed, he openly acknowledges, “I have a sort of fellow feeling with, with any veteran,” which suggests that service history is, for Matthew, the most important factor on which to judge their moral worth.

It is also interesting to note that the caseworkers, for the most part, consider their interactions with the prisoners in social terms, attributing positive characteristics, or “goodness,” to them on a personal level, too. James, for example, notes, “I don’t think I’ve come across anybody in prison I really don’t like, you know, to kind of socialize and chat.” This expression of warmth toward the prisoners is, again, demonstrative of James’s perception of them as individuals, but this time goes further than the classification of “ex-serviceman” rather than “prisoner”: he considers prisoners as people with whom he can “socialize,” as he would with his equals—including his former military colleagues. Moreover, that he seems to “like” all of the prisoners irrespective of their offense indicates that he can recognize some “goodness” in each of them on a fundamental, human level, something that mirrors Crawley’s (2004) suggestion that Prison Officers “who do. . .get to know the prisoners in their care may come to recognize their virtues as well as their frailties and, in consequence, find that they get to like some of the prisoners too” (p. 94). This is particularly important to highlight given the existence of negative preconceptions about those in prison: as Matthew admits, “you might think the people who are locked up would be morose and ag- even aggressive and fed up and so on, but I’ve hardly ever met that.” Although Matthew acknowledges that prisoners are expected to be “aggressive,” he presents his experience as resisting public perceptions of risk and dangerousness, reflecting the attitude of caseworkers and their attempt to construct an alternative individually assessed view, which departs from the generalized notion and image of the prisoner.

### Crime as a Mistake or Misfortune

It was also possible to identify a common perception among caseworkers that the crimes committed by the prisoners within their caseload were not deliberate, active decisions to break the law but rather “mistakes”: the unfortunate consequences of a lapse in judgment or a poor choice, often due to difficult circumstances which rendered no apparent alternative for them. Some of the caseworkers, including Matthew, frame this idea in terms of misfortune:

*They’ve had often very difficult childhoods. Um, and they’ve had all the, the, the bad luck in life. Things really gone against them and they’ve re-arrived in situations which, um, you know, they’ve made the wrong sort of decisions and the wrong moral choices. M- But thinking about it, if one had been exactly in their shoes, er, would one have been any different?*

Although Matthew acknowledges that the decisions of the prisoners have been “wrong,” his use of the concept of “bad luck” and things having “gone against” the prisoners is perhaps indicative of a belief that the prisoners cannot be held entirely responsible for their actions; indeed, he suggests that there is little reason to believe that anyone, including himself, might have made a different choice under the same circumstances, reflecting his belief in the absence of criminal intent on the part of the prisoner. Wrongdoing, in this case, is presented as justified due to life experiences, and Matthew refers explicitly to the mitigating role of “very difficult childhoods.” Reflecting on this later, he suggests that the prisoners have “arrived in situations” such as imprisonment, suggesting that their pathways into crime were not necessarily an outcome of individual choice but the natural destination of adverse life journeys. This is something that Paul also acknowledges when he speaks of his increased understanding of the “pitfalls, of what people come by and what, what really can and does go wrong for them”; the absence of active voice highlights the reluctance in attributing agency and intent for committed crimes and subsequent imprisonment; instead, such actions are presented within a fatalistic framework, whereby prisoners are almost passive participants in their life trajectories.

It is interesting to consider this idea in relation to the caseworkers’ motivations for working with these prisoners. Being able to rationalize a prisoner’s past decisions (and thus criminal behavior) as a consequence of misfortune not only allows the caseworkers to perceive them as being able to follow a different path in the future, if their circumstances can be changed but also to attribute their own lives to good fortune, and thus work to redress the balance of “luck.” As Matthew eloquently explains,

*I feel it in a way it’s sort of payback time, um, and a, it’s a little bit like, you know, there’s a reason why people who’ve earned lots of money should pay quite a bit of tax to help people who, er, you know, need, er, some help-* . . .*financial benefits and so on and it’s a bit like that, it’s that, er, one being in a very, you know, privileged position, as it were, to have, to have had good luck in life and things have gone well and, and here are people who, where, things have really gone bad for them in a variety of ways.*

This idea of being in a “privileged position” in comparison to the prisoners is particularly notable, as it suggests Matthew does not attribute his circumstances to anything he himself has done; instead, he considers his relative position of success as a result of his having “good luck in life” and “things hav[ing] really gone bad” for the prisoners. It is perhaps for this reason that Matthew considers it his duty to equalize his fortunes: he feels he “should” “payback” his excess of “good luck in life,” irrespective of the individual actions of the prisoners, because he is aware that, under different circumstances, he could be the one imprisoned. Viewing the prisoners through this lens, then, enables the caseworkers to place a greater value on the impact of their work: Richard assumes that prisoners, too, have the capacity to have a “reasonably successful, happy life” if their fortune is balanced and, if so, they may pay this back in the future. Changing the prisoners’ circumstances, therefore, not only impacts them as individuals but also, as Paul points out, “on a sort of societal level, I think um, the, um, levels of, um, reoffending are terrible and I think they should, um be brought down and if I can contribute in a small way toward that, I will.” This suggests that, for Paul, the responsibility for change cannot lie with the individual prisoner alone but instead demands an approach that recognizes and addresses the structural and interactional contributors to incarceration.

The ability to frame crimes as mistakes also allows the caseworkers to minimize the danger of their working environment and the people they encounter during their visits. In particular, the majority of the caseworkers compare the prison environment to other, perceptually more dangerous environments that they have experienced in order to draw a positive comparison: James explains that, “in my professional life, I’ve worked a lot with very severely mentally ill people, um, who were probably much more dangerous”; Richard highlights that, “you’re probably much safer inside the prison where there’s prison staff within sight everywhere you go,” and Matthew goes as far as to suggest a “rather imaginary risk aspect” of working in prisons, given his experience of “risky situations, far more risky than one would ever be in prison.” Although the caseworkers all temper their statements, using words such as “probably” and “rather” as an estimation, it appears important for them to reassure themselves about their personal safety within the prison environment;. At the same time, they acknowledge that it is not entirely safe; mitigating factors, including the implicit understanding that they are interacting with inherently “good” prisoners who did not intend to commit a crime, mean it is easier for the caseworkers to perceive the prisoners as rational people, rather than “criminals.” This may be particularly important for first-time or inexperienced caseworkers, who, as Paul notes, have “the inevitable apprehension of actually being in a prison” but “can’t be scared, I mean, you’ve got to just go for it, I mean, as in, if you’re scared, don’t go through the first door because it’s prison and um, you’ve got to, you know, accept the environment of the prison.” In admitting his own nervousness here, through his use of “you,” Paul demonstrates his understanding that caseworkers are among a few people who experience prison at will, and have to rationalize this decision; the inference that there is almost no going back “through the first door” implies that there is a risk involved, but one which he has considered, and accepted. It is interesting to note that this rationalization of the prison environment and the people within it is also a common practice among Prison Officers, another group who have made a conscious decision to be part of the prison environment. One Prison Officer interviewed about this stated,

A lot of people come into the job and they’ve got this view of prisoners, and it doesn’t help. . . Some of them see inmates as the lowest of the low—as the scum of the earth. But you can’t tar them all with the same brush. . . Some of them are very dangerous people, but some of them are okay. You try and do what you can for them. (Crawley, 2004, p. 107)

Through acknowledging that “some of them are okay,” this Prison Officer, too, demonstrates that he is mindful that the prisoners are also individuals and that separating “the person” from “the criminal” in this way makes him more able and willing to “do what [he] can for them” to make life a little easier. Although it is clear that not all Officers feel this way, and indeed, he is more explicit than the caseworkers in his recognition that “some of them are very dangerous people”—an understandable perspective, perhaps, given his more punitive role—it is clear that perceiving prisoners as separate from their crimes is imperative for those who seek to work toward rehabilitation rather than punishment.

### Compassion and the Shared Obligation to Support Ex-Servicemen in Prison

The second theme identified during data analysis was that of compassion, defined as an understanding of the emotional state of others, combined with a desire to alleviate their suffering. By putting themselves, metaphorically, in the position of the prisoners, the caseworkers empathize with the emotional toll of imprisonment and believe that, irrespective of their crimes, the prisoners are deserving of the caseworkers’ help. There was also a strong belief among the caseworkers that the prisoners in their caseload have “earned” the right to be looked after due to their military commitment. Matthew introduces this idea when he notes,

. . . *if they’ve had very short service, you know, sometimes you feel if the chap’s flunked out of training after seven months or something, which often they have, um, you know, why are we bothering sort of thing and I think well, actually, when you, when you sign on, as it were, when you’re attested and, and, and, and make your oath to the Queen and so on and so forth and and sign below as it were and get your Queen’s shilling, you’re actually saying, you know, I’m prepared to be sent to somewhere really nasty and be shot at*. . .*and have a miserable time*. . .*on behalf of the nation. And, and so they, every one of them has made a commitment.*

In acknowledging that all of the prisoners have at some stage “made a commitment” to risk their lives and “be shot at” if required “on behalf of the nation,” Matthew distinguishes between those who have made this “oath to the Queen” and “everyone else.” In doing so, it appears that Matthew elevates the prisoners’ “commitment” above all of their subsequent misdemeanors: it is the sole answer to his question, “why are we bothering” which suggests that he considers the prisoners as first and foremost “ex-servicemen” and it is this inalienable classification that has earned them the right to assistance. Matthew’s use of the first person “I’m” suggests that he recognizes his own service background and commitment alongside the prisoners’ and is, perhaps, indicative of an element of the personal investment he feels in the care of his fellow ex-servicemen: he has a true insight into the consequences of the prisoners’ “commitment” and, therefore, of its enduring significance and the worthiness of those who made it. It is, perhaps, for this reason that Matthew makes clear that his statement applies “even if they’ve had very short service”: the reality of having to be “sent to somewhere really nasty” is almost irrelevant, as it is the prisoners’ willingness to sacrifice themselves—the commitment, rather than the experience—that affords them the classification of “serviceman.” Matthew also demonstrates this through his references to “the Queen,” indicating his belief that the prisoners have made their “commitment” at the highest level; for him, then, it is expected that those who are served bear a reciprocal responsibility to look after those who would have made the ultimate sacrifice on their behalf, “the Queen’s shilling” serving to illustrate this concept.

Of course, it is essential to recognize that caseworkers without a service background also place great importance on the prisoners’ military connection: in explaining the motivations for their roles, Paul recalls that “I just knew that I really wanted to. . .support veterans,” and James states, “it is because they’re ex-servicemen and at the moment they find themselves in prison.” In fact, James’ certainty that the “ex-service” connection is at the heart of his role is repeated throughout his transcript, as he states both that he will visit a prisoner “just because they’re in there and they’re ex-service” and that the ex-servicemen prisoners are “lucky, if you like, that they’ve got the service history so that we can get involved because. . .there must be lots more people in there, that have got nobody.” While there might be a suggestion here that prisoners who are not ex-servicemen are less deserving of the caseworkers’ help, this distinction can also be seen as necessary for James to maintain the boundaries of his role toward the specific population: his reflection that there are “lots more people” in prison who need help but “have nobody” demonstrates that he is aware that there are imprisoned individuals who are deserving of support. His stance, in this case, is indicative of the sense of relatedness and responsibility due to the shared past with the ex-servicemen, which appears to be a motivating factor behind his role, rather than a judgment toward prisoners with different life experiences.

With all this in mind, it is interesting to note that James suggests that the prisoners “find themselves in prison,” as if without agency, particularly as this is reminiscent of the idea that imprisonment itself “infantilizes” people (Williams, 1996, p. 189 and, for a further example, Crawley, 2004, p. 131). The idea that the prisoners have no responsibility for themselves may appear incongruous with the notion of the ex-serviceman, particularly held by Matthew (although, of course, it can be argued that individual agency is not advocated in the Armed Forces); however, it is important to consider that, running parallel to this concept, is the notion that the prisoners are deserving of help because being in the Forces has damaged them in some regard. As Charlie explains, “at the end of the day, as a Forces community, they have been very damaged quite apart from whatever they’ve done against civil society or other serving people. . .I think we have a duty to all these people.” Here, Charlie implies that those serving in the Armed Forces experience emotional or mental distress and that this in itself is enough of a reason to offer the prisoners assistance—“quite apart from whatever they’ve done” to result in their imprisonment. In this way, Charlie also suggests that it is membership of the “Forces community” that is of importance: he does not consider the prisoners’ crimes to affect this categorization, nor does he, it appears, need evidence that the prisoners themselves have been “damaged” past their imprisonment. Of course, this suggests that Charlie perhaps considers the prisoners’ crimes to be a consequence of this damage, reinforcing his belief that there is a shared obligation—“we have a duty”—to help ex-servicemen escape circumstances they could not have avoided.

## “In” Versus “Out” of the Prison System

Some of the caseworkers perceive themselves to be unrestricted by the established norms associated with the criminal justice “system”: despite operating inside the prison environment and engaging with the prison population, Matthew considers the role of a caseworker as

*outside the legal system of their solicitors and things, you’re outside the prison system of, er, er, of their wing prison officers, you’re outside the sort of medical, mental health system, you’re completely, you know, out, an outsider as it were and that’s, I think, refreshing for the [prisoners].*

With no expectations upon him to change or manage the prisoners’ behavior, discipline or punishment but, instead, to assist however he can, Matthew is able to approach each prisoner as “an outsider,” without prejudice, and allow the prisoner the power to determine how he is perceived. In this way, both parties arguably become more exposed: the prisoner is free to reveal himself as an individual “untainted” by his crime or the expectations of the prison system, and Matthew must allow himself to trust that the prisoner is presenting his authentic self—or at least the version of himself that he wishes to be. Matthew’s acknowledgment that this is a “refreshing” approach for the prisoners further highlights his underlying perception that the “wing prison officers,” the “mental health system,” and even “the prison system” as a whole operate with an inherent bias: the assumption that the prisoners are at all times against the establishment, and must be controlled. Although “it would certainly be inaccurate to argue that the character of the staff-prisoner relationship is solely a relationship of ‘Them and Us’” (Crawley, 2004, p. 106), it is important to recognize that Prison Officers are required to maintain a certain level of mistrust and suspicion in order to remain vigilant and, therefore, cannot be expected to take prisoners at face value as the caseworkers can. Of course, it is also important to recognize that there is a fundamental difference between the functions of caseworkers and Prison Officers, which affect their perceptions of the prisoners. While Prison Officers are ‘obliged to regulate prisoners’ behavior (Crawley, 2004, p. xi), caseworkers are, in Paul’s words, “seldom, if ever, going to give [the prisoners] bad news”: the behavior of the prisoners does not impact upon the caseworkers’ and ensure that they do not escape’ ability to perform their role or their desire to do so. It, therefore, stands to reason that prisoners will perceive the caseworkers as an almost exclusively positive presence and, in the mind of the caseworkers at least, have little more to gain from their interactions by attempting to manipulate them. Therefore, while Prison Officers must “learn to manage feelings of sympathy and their natural inclination to help prisoners in their efforts to avoid being manipulated or ‘conditioned’ by prisoners” (Crawley, 2004, p. 147), caseworkers, removed from these pressures by being “outside the prison system,” are able to focus solely on their inclination to help and desire to alleviate suffering.

This is something that Charlie, in particular, feels is a pivotal part of his role, stating at length that it is,

*a question of being, perhaps being able to be a little bit er, able to be objective about things because you’re neither in the Forces neither are you in the Prison Service, or any of the- the Probation Service or anything like that, you haven’t got a job to do, but you are just a well-meaning person who, you know. . .just prepared to look at the thing as an overall situation and see if there’s any way you can provide information or make a link or, um, you know, do something to help encourage people to actually, you know, see that there is good in life and in people and try and be a little bit more trusting again, because I’m sure they must lose an awful lot of trust during the process of going to jail and then being in jail must be really, really soul-destroying. . .*

Charlie’s declaration that he is “just a well-meaning person” who “wants to do something to help” is demonstrative of an explicit separation of his caseworker role from the formal “services” previously encountered by the prisoners; it appears that Charlie perceives himself outside the boundaries of any establishment, as someone who has a unique role to play in restoring the prisoners’ faith in the goodness “in life and in people.” This suggests that Charlie considers institutions such as “the Forces” and “the Prison Service” as part of the reason that this role is required; his statement that the prisoners “must lose an awful lot of trust during the process” is indicative of his belief that the “services” do not treat people as individuals, but rather as “bodies that must be fed, brought from reception, got ready for court and so on” (Crawley, 2004, p. 153), making the experience of imprisonment one of dehumanization, too. Charlie’s recognition that the caseworkers “haven’t got a job to do” suggests that he is aware that fulfilment of operational objectives must take precedence for these institutions, but it also highlights that there are no such objectives for him: as such, Charlie *chooses* to invest in the prisoners’ emotional wellbeing because he deems it a worthwhile undertaking, in a way that is, perhaps, impossible for those who must maintain order among the prison population. Charlie’s perception that he is “objective” is, therefore, particularly interesting, as it suggests he considers objectivity as the absence of legal or moral judgments about the individual prisoners, rather than the consistent processes by which “the Prison Service” is able to manage the prison population as a whole. This idea is reinforced by Charlie’s use of “soul-destroying” to describe the experience of incarceration, an emotive phrase which suggests that the prisoners may be so damaged by their imprisonment that he *must* strive to demonstrate that there is some “good” left in the world. For Charlie then, not only are the prisoners valuable people, who are worthy of believing in goodness, but also damaged people, whose souls need mending, regardless of their criminal behavior.

Of course, this is demonstrative of the caseworkers’ perception that kindness is a more effective means of reducing recidivism than punishment: as Charlie makes clear, “we can’t shun our responsibilities to people just because they’ve done, you know, hopefully, they’ve paid the price, they’ve paid their dues and. . .hopefully . . .the better they’re treated the more likely they are to stay on the straight and narrow.” Richard summarizes the caseworkers’ perspective when he states, “I think, in some ways, [the caseworkers’ organization] helps dignify some people who’ve fallen pretty low,” later elaborating, “I think we. . . [care] for the fallen, you know, fallen from grace.” Richard’s repeated use of the word “fallen” here evokes a sense of accidental displacement, as if from a higher position, demonstrating his perspective that the prisoners were once recognized as valuable citizens, a social position that can be restored. That Richard chooses to use the word “grace,” with its connotations of beauty, elegance and refinement, reinforces this perspective, attributing qualities to the prisoners as people that were replaced with the label, “criminal.” Indeed, such attributes are arguably among the most valued in society, reflecting, perhaps, the high esteem in which members of the Armed Forces are traditionally held. Through “dignifying” the prisoners again, the caseworkers aim to overturn this labeling, demonstrating to the prisoners that they did, do, and will have value as individuals, removed from, and in spite of, their crime and the consequences thereof. Through their actions, then, the prisoners can come to see themselves as the caseworkers see them and be “saved” from the consequences and cause of the “criminal” label.

## Discussion

Although, as Tomczak and Albertson (2016) argue, “punitive discourses and practices can never be absent from custodial settings” (p. 58), the caseworkers’ transcripts support the notion that volunteers’ perceived and actual separation from the disciplinary aspects of the Prison Service affords them the distinct opportunity to conceptualize prisoners as “people” rather than “offenders.” This, in turn, allows them to form quantifiably different relationships with the prisoners, as compared to the Prison Officers, who “can too easily lose sight of the ‘people’ serving the sentences” (Tomczak & Albertson, 2016, p. 63), focused as they are on maintaining order and discipline in a fraught and often chaotic environment. That the caseworkers, too, are able to maintain a non-judgmental approach toward the prisoners is reflective of their belief in the inherent potential for goodness in each of them, something that is demonstrated by the prisoners’ status as “ex-servicemen” and the high regard in which this is held by the caseworkers. Thus, the caseworkers provide further evidence for the existence of a distinctive “voluntary sector ethos of compassion” (Tomczak & Albertson, 2016, p. 65), aligning closely with Tomczak and Albertson’s findings that relationships with voluntary practitioners can have an enduring impact on a prisoner’s experience of imprisonment and hope for the future. This, of course, suggests there are means of improving prisoners’ experience while imprisoned and strengthens Liebling et al.’s (2015) argument that relationships constitute “the quality of prison life” (p. 59). Indeed, in building relationships based on mutual respect, on a human level, the caseworkers demonstrate the potential for approaching prisoners as individuals to transcend the “criminal” label, allowing, perhaps, the prisoners to redeem a sense of “self” and move toward feeling worthy of rehabilitation and a good life.

It is particularly interesting to consider this in relation to the popular Risk-Need-Responsivity model, which states primarily that “treatment should focus on criminogenic needs (i.e., those needs empirically associated with recidivism reduction” (Gannon & Ward, 2014, p. 436), and is “widely regarded to be the received or orthodox position concerning rehabilitation” (Gannon & Ward, 2014, p. 436), arguably due to its simplicity, cost-effectiveness and focus on “risk-reduction which resonates well with the security-oriented culture of correctional establishments” (Gannon & Ward, 2014, p. 436). Against a backdrop of rapidly escalating prison populations, with the Prison Service facing extreme pressure to manage the burgeoning crisis of offender management, it is clear that effective treatment is of paramount importance; however, given that even Prison Officers “are under no illusions that prison will deter prisoners from further crimes” (Crawley, 2004, p. 96) it is, perhaps, pertinent to consider the evidence that supports individually tailored treatment in the management of problem behaviors (Gannon & Ward, 2014, p. 438). With current practice focusing almost exclusively on offending behavior, the “human factors” so valued by the caseworkers and demonstrated through their compassion toward the prisoners are forgotten: prisoners are once again reduced to the sum of their crimes. The caseworkers’ experiences, therefore, offer tentative support to Gannon and Ward’s (2014) argument that the focus of rehabilitation should be shifted toward Evidence-Based Practice, in which behavior change is enabled through establishing relationships based on “respectfulness and factors related to genuineness (i.e., empathy, warmth, openness, trustworthiness” (p. 443)—or, in other words, compassion. It is unsurprising that the Good Lives Model relates so well to the caseworkers’ experience, grounded, as they both are, in the fundamental principle of human dignity and inalienable rights (see Ward & Syversen, 2009), as opposed to the stereotypical image of the prisoner proliferated by politicians and the media, in which those sent to prison “may not just be presented as predominantly violent, but as ‘hardened criminals’” (Warner, 1998, p. 122).

It is also important to keep in mind that volunteers, although no longer “derided as naive ‘do-gooders’, within the prison system [may still] be viewed with considerable suspicion by criminal justice staff” (Mills et al., 2012, p. 394), particularly those who believe they are opposed to the work of the prison; the role of an individual volunteer is far removed from that of a professional third sector organization, whose “appeal as partners in service provision [is] well-rehearsed, and include[s] their specialist expertise, and cost-effectiveness” (Mills et al., 2012, p. 392) and, therefore, must be considered as a separate undertaking, and one which could, perhaps, be viewed as an interference to the main function of the prison. It is, therefore, important not to consider the caseworkers’ approach as a perfect solution to the problems of the Prison Service but rather to think critically about their position: without performance objectives, business-critical targets, or, in fact, formal responsibility for the prisoners’ rehabilitation, it is, perhaps, easier to remain optimistic about the prisoners’ prospects and to invest time and resources in compassion, rather than convenience. After all, there *are* risks to manage in any prison population, and, with the Prison Service in a series of crises (Justice Committee, 2019), it is possible that the caseworkers’ perspective could be viewed as a disruptive force within an already precarious institution, albeit one which does not necessarily instigate structural change. Similar research has, for example, argued that despite the clear benefits of volunteer schemes, such as the Listeners for the prisoners and prison environment, they might be “perpetuating unjustifiable justices and harms” by unintentionally deflecting attention from institutional shortcomings and, in a way, halt criminal justice reform (Tomczak & Bennett, 2020, p. 647). Our research has made a preliminary contribution to the task of understanding the complex and multifaceted relationships between caseworkers working with a specific population and the Prison Service; future work should focus on interrogating these relationships and the work of volunteers within the larger systems in which volunteers operate.

Furthermore, it is important to reiterate that the experiences of these Prison In-Reach Caseworkers are necessarily restricted by the clients with whom they work: all of the prisoners are ex-servicemen, and thus the relationships between the prisoners and caseworkers are framed, facilitated and constrained by this distinct trait presentation and attitudinal response. That the caseworkers revealed, as Albertson et al. (2017) have argued, “‘veterans’ offending is more appropriately positioned amongst wider structural challenges faced on return to civilian society” (p. 23), confirms that this consideration has a significant influence on their perspective; that “contact with the criminal justice system represents just one of a myriad of *harms* that may be experienced by individuals who leave the Armed Forces” (Albertson et al., 2017, p. 24, emphasis added) suggests that ex-servicemen may be primarily perceived as vulnerable individuals, and thus disproportionately in need of specific and individually tailored support. The caseworkers’ feeling of obligation toward the prisoners because of their classification as ex-servicemen also chimes with McGarry and Walklate’s (2015) framework for understanding their engagement in crime: by “imagining the ‘soldier as victim’, [they] assert that the state is accountable for exposing military personnel to combat, which can have a detrimental impact on their return to civilian life” (Albertson et al., 2017, p. 24), and it follows that the state is accountable for alleviating the resulting distress. The caseworkers emphasize it is the commitment to experience combat, rather than the experience itself, that matters and remains the prisoners’ defining characteristic; thus, the caseworkers’ experience supports Banks and Albertson’s (2018) assertion that criminal justice policymakers and practitioners must consider the distinct experiences and needs of ex-servicemen if arrest, imprisonment and reoffending are to be reduced.

At this stage, it is useful to consider the caseworkers’ experiences in relation to Becker’s (1963) suggestion that “deviance is *not* a quality of the act the person commits, but rather a consequence of the application by others of rules and sanctions to ‘an offender’. The deviant is one to whom that label has been successfully applied” (p. 9). The transcripts reveal that Social Labeling Theory is at the heart of the caseworkers’ experience, particularly the conviction that the social identity of “criminal” is often adopted alongside a condemnation narrative (Maruna, 2001), which, in turn, elicits a sense of hopelessness about the prisoner’s prospects for redemption, both for the individual and the society into which they are, or hope to be, released. As Menninger argues, “once someone has been labelled an offender. . .he is fair game, and our feelings come out in the form of a conviction that a hurt society should be “repaid” (Menninger, 1969, p. 190); this, in practice, equates to a desire not to let the “criminal” forget that he has committed a crime, and thus forego his claim to be recognized as a “fully-fledged, *trusted* member of society” (Sykes, 2007, p. 66). By contrast, through reaffirming the prisoner’s social identity as “ex-serviceman,” the caseworkers can help to rewrite a prisoner’s narrative using a redemption script which, in maintaining the essential goodness of the prisoner, can be internalized to redeem their past and claim a meaningful future (Maruna, 2001). This, in turn, enables the prisoner to feel hopeful about their future prospects and chances of avoiding reoffending: the basis of desistance, as conceptualized as “a process of maintaining crime-free behavior in the face of life’s obstacles and temptations” (Sundt, 2010, p. 575).

It is essential to recognize that for the caseworkers, this positive social identity, or label, is absolute: regardless of their crimes, the ex-servicemen remain ex-servicemen first, which chimes with Sundt’s (2010) understanding that the redemption script is founded on embracing an earlier identity—“not the ‘new me’, but the ‘real me’” (p. 576)—which is fundamentally “good.” This, in turn, suggests that social identity labels bear moral weight, too, something that is particularly poignant to consider in relation to the highly-stigmatized ‘sex offender’ label, especially given the widely-publicized finding that ex-servicemen who offend are significantly more likely to engage in violent and sexual offending than their civilian counterparts (MacManus et al., 2013). That the caseworkers are able to elevate ‘ex-serviceman’ above the ‘sex offender’ label, one that generates profound moral exclusion, even from other prisoners, and is often experienced as an assault on a prisoner’s ‘moral character’ (Ievins & Crewe, 2015, p. 482; see also Crawley, 2004, p. 99), demonstrates that the Armed Forces are held in, perhaps disproportionately, high regard (Hines et al., 2015; Mahar et al., 2017), and that the caseworkers believe the ex-service population is somehow quantifiably different from other populations (perhaps, for some, because of their personal experience of the Armed Forces). This powerful realization adds further support to the caseworkers” perception that recognizing and privileging an individual’s service identity, certainly above their “criminal” identity, may successfully establish, or restore, their self-esteem and a desire to reintegrate. The caseworkers’ beliefs then, can be framed as a real-world demonstration of the social identity approach, in which belonging to a valuable or socially desirable group provides access to social resources alongside contributing to increased self-esteem (Albertson et al., 2015; Jetten et al., 2012). Moreover, the significance of carving out a fresh perception of oneself cannot be understated in the context of the prison environment: as Goffman (1961) suggests, the experience of imprisonment as a total institution can have a profound impact on a person’s self-image, to the extent of “personal defacement” (p. 29), even before additional moral judgments based on one’s crime are experienced. Thus, as Warner (1998) argues, the fundamental aim of any rehabilitation process must incorporate an element of “enabling prisoners to cope with the destructive effects of being incarcerated” (p. 121): for the caseworkers, this both begins and ends with the label “criminal.”

Finally, then, it is important to acknowledge that the negative labeling of prisoners has its roots in wider social patterns; indeed, “the stigmatizing of prisoners often goes hand-in-hand with an exaggerated view of the extent of crime and, in particular, an exaggerated sense of things being out of control” (Warner, 1998, p. 123). While referring to the “epidemic” of crime may be demonstrative of a step change in recognizing that there could be societal means of approaching and reducing offending behavior, it is first imperative that there is also a step change in societal perceptions of those who “offend”: as Mays (1967) argues, the “social structure not only influences behavior and attitudes, *it also embodies principles and ideas in the first place*” (p. 198). Just as it is accepted that, sooner or later, most prisoners are released back into the community, so too must our aim be “the reintegration of the temporarily suspended individual back into the mainstream of social life, preferably a life at a higher level than before, just as soon as possible” (Menninger, 1969, p. 265). In doing so, it is imperative to recognize, as the caseworkers do, that “this cohort, not wholly heroes, victims or villains”—not just “veterans” or “criminals”—has “a right to a balanced and sensitive approach” (Albertson et al., 2017, p. 28) to understanding their incarceration and rehabilitation, which moves beyond emphasizing the individual’s responsibility for change and instead considers how society might best support all of its citizens.
